# Crystal structure of ethyl 2-(2,4,5-tri­meth­oxy­phen­yl)quinoline-4-carboxyl­ate

**DOI:** 10.1107/S2056989015011706

**Published:** 2015-06-27

**Authors:** T. O. Shrungesh Kumar, S. Naveen, M. N. Kumara, K. M. Mahadevan, N. K. Lokanath

**Affiliations:** a Department of Chemistry, Kuvempu University, Jnanasahyadri, Shankaraghatta 577 451, India; b Institution of Excellence, University of Mysore, Manasagangotri, Mysore 570 006, India; c Department of Chemistry, Yuvaraja’s College, University of Mysore, Mysore 570 005, India; d Department of Studies in Physics, University of Mysore, Manasagangotri, Mysore 570 006, India

**Keywords:** crystal structure, quinoline, quinolone-4-ethyl carboxyl­ate, hydrogen bonding, C—H⋯O inter­actions

## Abstract

In the title compound, C_21_H_21_NO_5_, the dihedral angle between the quinoline ring system (r.m.s. deviation = 0.028 Å) and the tri­meth­oxy­benzene ring is 43.38 (5)°. The C atoms of the meth­oxy groups deviate from their attached benzene ring by −0.396 (2), −0.049 (2) and 0.192 (2) Å for the *ortho*-, *meta*- and *para*-substituents, respectively. The pendant ethyl chain is disordered over two orientations in a 0.527 (5):0.473 (5) ratio. A short intra­molecular C—H⋯O contact closes an *S*(6) ring. In the crystal, inversion dimers linked by pairs of weak C—H⋯O inter­actions generate *R*_2_^2^(6) loops. The dimers are linked by further C—H⋯O inter­actions to generate [1-10] chains.

## Related literature   

For background to quinolines and their properties, see: Beagley *et al.* (2003 ▸). For our work in this area, see: Pradeep *et al.* (2014 ▸); Shrungesh Kumar *et al.* (2015 ▸); Sunitha *et al.* (2015 ▸).
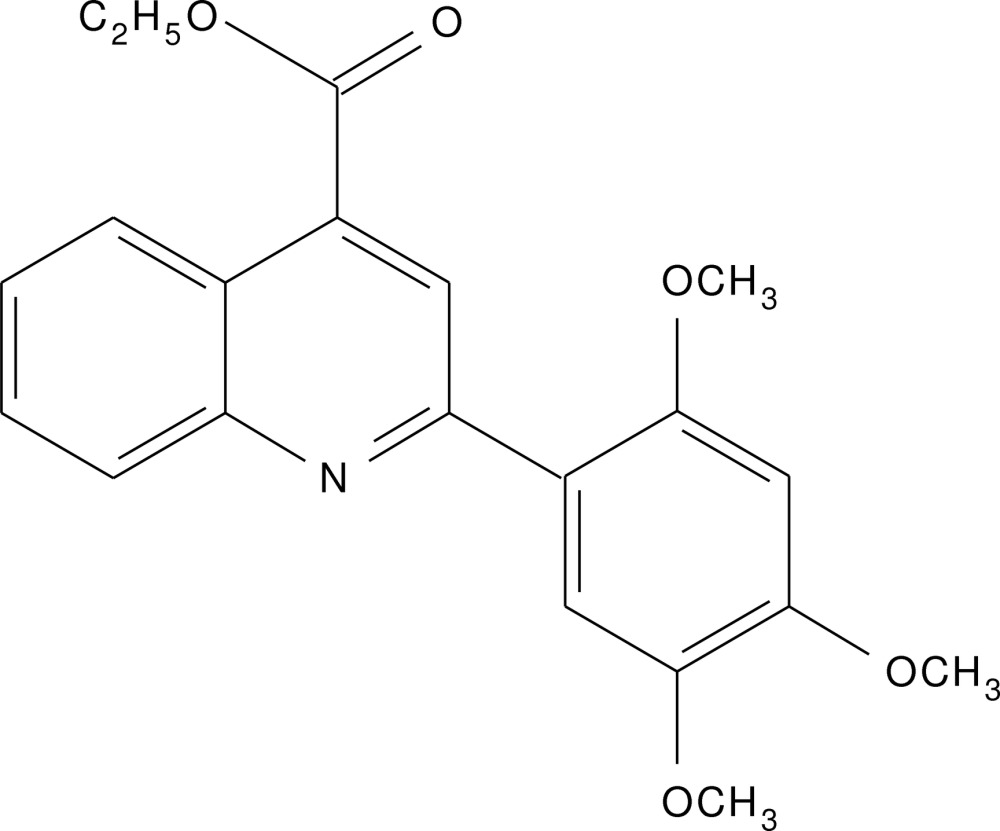


## Experimental   

### Crystal data  

C_21_H_21_NO_5_
*M* *_r_* = 367.39
Triclinic, 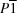

*a* = 8.3444 (3) Å
*b* = 9.3508 (4) Å
*c* = 12.2723 (5) Å
α = 104.079 (2)°
β = 97.282 (2)°
γ = 93.904 (2)°
*V* = 916.43 (6) Å^3^
*Z* = 2
Cu *K*α radiation
μ = 0.78 mm^−1^
*T* = 100 K
0.29 × 0.22 × 0.19 mm

### Data collection  

Bruker X8 Proteum diffractometer
Absorption correction: multi-scan (*SADABS*; Bruker, 2013 ▸) *T*_min_ = 0.797, *T*_max_ = 0.813
10087 measured reflections
3008 independent reflections
2601 reflections with *I* > 2σ(*I*)
*R*_int_ = 0.041

### Refinement  

*R*[*F* ^2^ > 2σ(*F* ^2^)] = 0.039
*wR*(*F* ^2^) = 0.117
*S* = 1.04
3008 reflections
277 parameters
H atoms treated by a mixture of independent and constrained refinement
Δρ_max_ = 0.20 e Å^−3^
Δρ_min_ = −0.28 e Å^−3^

### 

Data collection: *APEX2* (Bruker, 2013 ▸); cell refinement: *SAINT* (Bruker, 2013 ▸); data reduction: *SAINT*; program(s) used to solve structure: *SHELXS97* (Sheldrick, 2008 ▸); program(s) used to refine structure: *SHELXL97* (Sheldrick, 2008 ▸); molecular graphics: *Mercury* (Macrae *et al.*, 2008 ▸); software used to prepare material for publication: *Mercury*.

## Supplementary Material

Crystal structure: contains datablock(s) global, I. DOI: 10.1107/S2056989015011706/hb7449sup1.cif

Structure factors: contains datablock(s) I. DOI: 10.1107/S2056989015011706/hb7449Isup2.hkl

Supporting information file. DOI: 10.1107/S2056989015011706/hb7449Isup3.cml

**Fig. 1.**
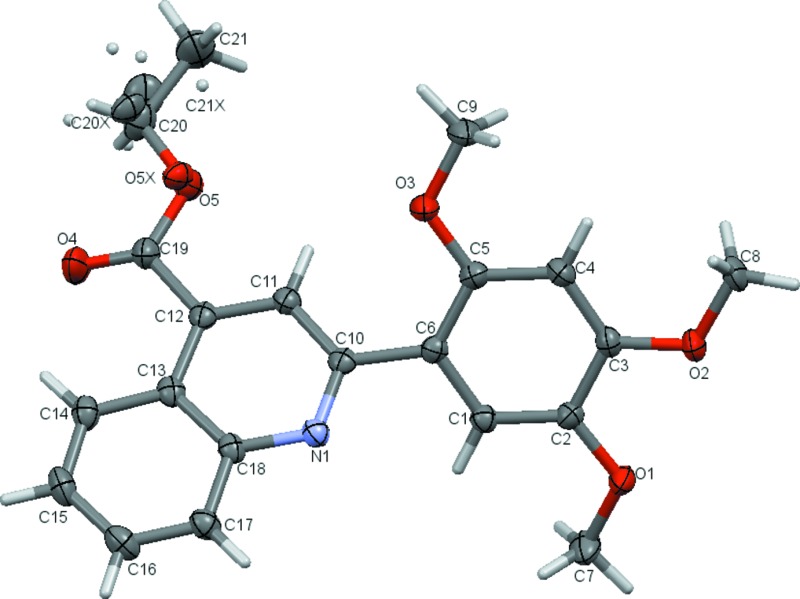
A view of the title molecule, with displacement ellipsoids drawn at the 50% probability level.

. DOI: 10.1107/S2056989015011706/hb7449fig1.tif
A view of the title mol­ecule, with displacement ellipsoids drawn at the 50% probability level.

CCDC reference: 1407284

Additional supporting information:  crystallographic information; 3D view; checkCIF report

## Figures and Tables

**Table 1:** Hydrogen-bond geometry (, )

*D*H*A*	*D*H	H*A*	*D* *A*	*D*H*A*
C14H14*A*O4	0.93	2.30	2.9073(18)	123
C9H9*A*O3^i^	0.96	2.53	3.397(2)	150
C20H20*A*O1^ii^	0.97	2.51	3.304(5)	139

Symmetry codes: (i) 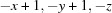; (ii) 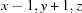.

## supplementary crystallographic information

### S1. Comment

Quinolines have been found to possess a wide spectrum of biological activities (Beagley *et al.*, 2003,). Among them, the quinolone-4-ethyl carboxylates have been identified as potent antagonizing agents. Keeping in view of their broad spectrum of medicinal properties and in continuation of our work on new quinoline based therapeutic agents (Pradeep *et al.*, 2014, Shrungesh Kumar *et al.*, 2015, Sunitha *et al.*, 2015), the title compound was synthesized. The compound obtained was characterized spectroscopically and its structure was established by X-ray crystallographic studies.

In the title compound, C_21_H_21_O_5_N, the carboxyl group is disordered. The two rings of the quinoline system are fused almost coaxially, with a dihedral angle between their planes of 2.66°. The dihedral angle between the quinoline ring system mean plane (r.m.s. deviation = 0.0238 Å) and the tri-methoxyphenyl ring is 43.34 (1)°. The structure exhibits both inter and intra-molecular hydrogen bonds of the type C–H···O.

### S2. Experimental

^1^H NMR was recorded at 400 MHz in CDCl_3_ solvent. Mass spectra were recorded on a Jeol SX 102=DA-6000 (10 kV) fast atom bombardment (FAB) mass spectrometer.

A mixture of 2-(2,4,5-trimethoxyphenyl)quinoline-4-carboxylic acid 1.0 g (0.005 mol) and absolute EtOH (15 ml) was stirred at 0–5°C. A catalytic amount of concentrated H_2_SO_4_ was added drop wise into the flask until the powdered 2-(2,4,5-trimethoxyphenyl)quinoline-4-carboxylic acid was dissolved. The solution was then refluxed for 15–17 h. The completion of the reaction was monitored by TLC [hexane and ethyl acetate (9:1 *v*/*v*)]. The reaction mixture was poured into a crushed ice (100 ml). The precipitate was collected by filtration, washed with water and EtOH, dried under vacuum to obtain the crude product in 85% yield. The crude product was purified by column chromatography using silica gel (60–120 mesh, petroleum ether: ethyl acetate, 9:1 *v*/*v*). Colourless rectangular crystals grew after 4 days due to slow evaporation of the solvent. Yield = 85%. M·P. = 100–102 °C.

### S3. Refinement

The hydrogen atoms were fixed geometrically (C—H= 0.93–0.96 Å) and allowed to ride on their parent atoms with *U*_iso_(H) =1.5*U*_eq_(*C*-methyl) and = 1.2*U*_eq_(C).

### Crystal data

**Table:**

C_21_H_21_NO_5_	*Z* = 2
*M**_r_* = 367.39	*F*(000) = 388
Triclinic, *P*1	^1^H NMR(400 MHz, CdCl_3_ ): δ = 8.71 (d, J = 8.40 Hz, 1H), 8.52 (s, 1H), 7.76 (t, J = 7.60 Hz, 1H), 7.63 (t, J = 6.00 Hz, 2H), 7.25 (s, 1H), 6.64 (s, 1H), 4.52 (q, J = 6.80 Hz, 2H), 3.97 (d, J = 5.20 Hz, 6H), 3.88 (s, 3H), 1.47 (t, J = 7.20 Hz, 3H) ppm. MS (70 eV) *m/z*(%): 368.0(M^+^).
Hall symbol: -P 1	*D*_x_ = 1.331 Mg m^−^^3^
*a* = 8.3444 (3) Å	Cu *K*α radiation, λ = 1.54178 Å
*b* = 9.3508 (4) Å	Cell parameters from 2601 reflections
*c* = 12.2723 (5) Å	θ = 6.9–64.4°
α = 104.079 (2)°	µ = 0.78 mm^−^^1^
β = 97.282 (2)°	*T* = 100 K
γ = 93.904 (2)°	Rectangular block, colourless
*V* = 916.43 (6) Å^3^	0.29 × 0.22 × 0.19 mm

### Data collection

**Table:**

Bruker X8 Proteum diffractometer	3008 independent reflections
Radiation source: Bruker MicroStar microfocus rotating anode	2601 reflections with *I* > 2σ(*I*)
Helios multilayer optics monochromator	*R*_int_ = 0.041
Detector resolution: 18.4 pixels mm^-1^	θ_max_ = 64.4°, θ_min_ = 6.9°
φ and ω scans	*h* = −9→9
Absorption correction: multi-scan (*SADABS*; Bruker, 2013)	*k* = −10→10
*T*_min_ = 0.797, *T*_max_ = 0.813	*l* = −14→14
10087 measured reflections	

### Refinement

**Table:**

Refinement on *F*^2^	Primary atom site location: structure-invariant direct methods
Least-squares matrix: full	Secondary atom site location: difference Fourier map
*R*[*F*^2^ > 2σ(*F*^2^)] = 0.039	Hydrogen site location: inferred from neighbouring sites
*wR*(*F*^2^) = 0.117	H atoms treated by a mixture of independent and constrained refinement
*S* = 1.04	*w* = 1/[σ^2^(*F*_o_^2^) + (0.0733*P*)^2^ + 0.1234*P*] where *P* = (*F*_o_^2^ + 2*F*_c_^2^)/3
3008 reflections	(Δ/σ)_max_ < 0.001
277 parameters	Δρ_max_ = 0.20 e Å^−^^3^
0 restraints	Δρ_min_ = −0.28 e Å^−^^3^

### Special details

**Table:**

Geometry. Bond distances, angles *etc*. have been calculated using the rounded fractional coordinates. All su's are estimated from the variances of the (full) variance-covariance matrix. The cell e.s.d.'s are taken into account in the estimation of distances, angles and torsion angles
Refinement. Refinement on *F*^2^ for ALL reflections except those flagged by the user for potential systematic errors. Weighted *R*-factors *wR* and all goodnesses of fit *S* are based on *F*^2^, conventional *R*-factors *R* are based on *F*, with *F* set to zero for negative *F*^2^. The observed criterion of *F*^2^ > σ(*F*^2^) is used only for calculating -*R*-factor-obs *etc*. and is not relevant to the choice of reflections for refinement. *R*-factors based on *F*^2^ are statistically about twice as large as those based on *F*, and *R*-factors based on ALL data will be even larger.

### Fractional atomic coordinates and isotropic or equivalent isotropic displacement parameters (Å^2^)

**Table:**

	*x*	*y*	*z*	*U*_iso_*/*U*_eq_	Occ. (<1)
O1	0.85573 (12)	−0.06460 (10)	0.25750 (8)	0.0282 (3)	
O2	0.82233 (13)	−0.11123 (10)	0.03839 (8)	0.0292 (3)	
O3	0.55682 (13)	0.33976 (11)	0.05983 (8)	0.0315 (4)	
O4	0.22604 (13)	0.74517 (12)	0.41032 (9)	0.0388 (3)	
O5	0.1869 (4)	0.5760 (4)	0.2445 (2)	0.0279 (8)	0.527 (5)
N1	0.73685 (14)	0.47119 (12)	0.39148 (9)	0.0220 (3)	
C1	0.73786 (16)	0.17037 (14)	0.27663 (12)	0.0211 (4)	
C2	0.78484 (16)	0.04140 (15)	0.21340 (12)	0.0215 (4)	
C3	0.76438 (16)	0.01469 (15)	0.09451 (12)	0.0228 (4)	
C4	0.68822 (17)	0.11314 (15)	0.04271 (12)	0.0247 (4)	
C5	0.63437 (16)	0.24009 (15)	0.10755 (12)	0.0226 (4)	
C6	0.66258 (16)	0.27241 (14)	0.22587 (12)	0.0203 (4)	
C7	0.8450 (2)	−0.05532 (17)	0.37384 (13)	0.0361 (5)	
C8	0.81347 (19)	−0.13690 (17)	−0.08181 (12)	0.0315 (5)	
C9	0.5312 (2)	0.31046 (18)	−0.06125 (12)	0.0370 (5)	
C10	0.62512 (16)	0.41161 (14)	0.30220 (11)	0.0204 (4)	
C11	0.47701 (17)	0.47445 (15)	0.28373 (12)	0.0222 (4)	
C12	0.44526 (17)	0.59953 (14)	0.35868 (11)	0.0218 (4)	
C13	0.56525 (16)	0.67030 (14)	0.45409 (12)	0.0222 (4)	
C14	0.55434 (18)	0.80529 (15)	0.53426 (12)	0.0257 (4)	
C15	0.67839 (19)	0.86451 (16)	0.62120 (13)	0.0296 (4)	
C16	0.81946 (18)	0.79239 (17)	0.63317 (13)	0.0295 (5)	
C17	0.83567 (18)	0.66226 (16)	0.55755 (12)	0.0263 (4)	
C18	0.70992 (16)	0.59871 (14)	0.46574 (11)	0.0215 (4)	
C19	0.28323 (18)	0.65885 (16)	0.34077 (12)	0.0282 (4)	
C20	0.0230 (6)	0.6201 (5)	0.2213 (3)	0.0326 (12)	0.527 (5)
C21	−0.0515 (4)	0.5296 (4)	0.1068 (3)	0.0495 (13)	0.527 (5)
C20X	0.0784 (5)	0.6858 (5)	0.1962 (3)	0.0279 (11)	0.473 (5)
C21X	−0.0638 (6)	0.5817 (5)	0.2021 (4)	0.0389 (14)	0.473 (5)
O5X	0.2315 (4)	0.6296 (4)	0.2276 (3)	0.0255 (9)	0.473 (5)
H7A	0.89180	−0.13750	0.39500	0.0540*	
H7C	0.90300	0.03580	0.42020	0.0540*	
H8A	0.86270	−0.22540	−0.11110	0.0470*	
H7B	0.73310	−0.05810	0.38480	0.0540*	
H1A	0.75670	0.19040	0.35550	0.0250*	
H4A	0.67280	0.09450	−0.03610	0.0300*	
H9B	0.46750	0.21660	−0.09300	0.0550*	
H9C	0.63410	0.30780	−0.08850	0.0550*	
H11A	0.40110	0.43010	0.22010	0.0270*	
H14A	0.46170	0.85430	0.52770	0.0310*	
H15A	0.66930	0.95350	0.67290	0.0360*	
H16A	0.90240	0.83360	0.69310	0.0350*	
H17A	0.92940	0.61520	0.56630	0.0320*	
H20A	0.02880	0.72470	0.22330	0.0390*	0.527 (5)
H20B	−0.04160	0.60270	0.27810	0.0390*	0.527 (5)
H21A	−0.15830	0.55740	0.08850	0.0740*	0.527 (5)
H21B	−0.05870	0.42660	0.10630	0.0740*	0.527 (5)
H21C	0.01440	0.54650	0.05150	0.0740*	0.527 (5)
H8B	0.87000	−0.05420	−0.09920	0.0470*	
H8C	0.70180	−0.14830	−0.11600	0.0470*	
H9A	0.47470	0.38730	−0.08350	0.0550*	
H20C	0.07290	0.69820	0.11960	0.0330*	0.473 (5)
H20D	0.07340	0.78210	0.24710	0.0330*	0.473 (5)
H21D	−0.16300	0.61860	0.17740	0.0580*	0.473 (5)
H21E	−0.06240	0.57460	0.27900	0.0580*	0.473 (5)
H21F	−0.05690	0.48550	0.15380	0.0580*	0.473 (5)

### Atomic displacement parameters (Å^2^)

**Table:**

	*U*^11^	*U*^22^	*U*^33^	*U*^12^	*U*^13^	*U*^23^
O1	0.0405 (6)	0.0247 (5)	0.0224 (6)	0.0145 (4)	0.0067 (5)	0.0076 (4)
O2	0.0396 (6)	0.0251 (5)	0.0220 (6)	0.0129 (4)	0.0073 (4)	0.0004 (4)
O3	0.0467 (7)	0.0290 (6)	0.0194 (6)	0.0160 (5)	0.0014 (5)	0.0060 (4)
O4	0.0359 (6)	0.0413 (6)	0.0347 (6)	0.0193 (5)	0.0053 (5)	−0.0032 (5)
O5	0.0231 (14)	0.0331 (16)	0.0269 (14)	0.0105 (11)	0.0050 (10)	0.0040 (11)
N1	0.0230 (6)	0.0229 (6)	0.0196 (6)	0.0028 (5)	0.0038 (5)	0.0040 (5)
C1	0.0213 (7)	0.0225 (7)	0.0198 (7)	0.0027 (5)	0.0045 (6)	0.0050 (6)
C2	0.0209 (7)	0.0214 (7)	0.0228 (7)	0.0042 (5)	0.0029 (6)	0.0067 (6)
C3	0.0233 (7)	0.0207 (7)	0.0228 (8)	0.0031 (5)	0.0053 (6)	0.0012 (6)
C4	0.0283 (8)	0.0266 (7)	0.0180 (7)	0.0038 (6)	0.0031 (6)	0.0036 (6)
C5	0.0229 (7)	0.0233 (7)	0.0219 (8)	0.0042 (5)	0.0021 (6)	0.0066 (6)
C6	0.0180 (6)	0.0220 (7)	0.0212 (7)	0.0023 (5)	0.0038 (5)	0.0055 (6)
C7	0.0576 (11)	0.0295 (8)	0.0277 (9)	0.0165 (7)	0.0121 (8)	0.0134 (7)
C8	0.0379 (9)	0.0317 (8)	0.0214 (8)	0.0095 (6)	0.0070 (6)	−0.0027 (6)
C9	0.0579 (11)	0.0331 (8)	0.0196 (8)	0.0135 (7)	−0.0025 (7)	0.0080 (7)
C10	0.0227 (7)	0.0215 (7)	0.0194 (7)	0.0034 (5)	0.0059 (6)	0.0082 (6)
C11	0.0239 (7)	0.0237 (7)	0.0193 (7)	0.0039 (5)	0.0033 (6)	0.0057 (6)
C12	0.0256 (7)	0.0219 (7)	0.0206 (7)	0.0054 (5)	0.0067 (6)	0.0083 (6)
C13	0.0261 (7)	0.0213 (7)	0.0211 (7)	0.0016 (5)	0.0086 (6)	0.0069 (6)
C14	0.0294 (8)	0.0236 (7)	0.0257 (8)	0.0046 (6)	0.0105 (6)	0.0054 (6)
C15	0.0375 (8)	0.0231 (7)	0.0258 (8)	−0.0009 (6)	0.0124 (7)	−0.0014 (6)
C16	0.0295 (8)	0.0333 (8)	0.0220 (8)	−0.0044 (6)	0.0054 (6)	0.0011 (6)
C17	0.0247 (7)	0.0309 (8)	0.0220 (8)	0.0018 (6)	0.0053 (6)	0.0036 (6)
C18	0.0249 (7)	0.0214 (7)	0.0194 (7)	0.0015 (5)	0.0079 (6)	0.0056 (6)
C19	0.0323 (8)	0.0288 (7)	0.0239 (8)	0.0113 (6)	0.0050 (6)	0.0046 (6)
C20	0.022 (2)	0.035 (2)	0.042 (2)	0.0086 (18)	0.0069 (19)	0.0095 (17)
C21	0.0295 (18)	0.057 (2)	0.052 (3)	0.0140 (15)	−0.0062 (16)	−0.0014 (19)
C20X	0.0195 (18)	0.034 (2)	0.033 (2)	0.0086 (15)	0.0046 (14)	0.0120 (16)
C21X	0.024 (2)	0.042 (2)	0.050 (3)	−0.0030 (19)	0.010 (2)	0.010 (2)
O5X	0.0205 (15)	0.0359 (18)	0.0222 (14)	0.0111 (12)	0.0051 (11)	0.0084 (12)

### Geometric parameters (Å, º)

**Table:**

O1—C2	1.3729 (17)	C17—C18	1.419 (2)
O1—C7	1.4238 (18)	C20—C21	1.485 (5)
O2—C3	1.3645 (17)	C20X—C21X	1.503 (7)
O2—C8	1.4272 (17)	C1—H1A	0.9300
O3—C5	1.3718 (18)	C4—H4A	0.9300
O3—C9	1.4291 (17)	C7—H7A	0.9600
O4—C19	1.1951 (18)	C7—H7B	0.9600
O5—C19	1.368 (3)	C7—H7C	0.9600
O5—C20	1.469 (6)	C8—H8A	0.9600
O5X—C20X	1.457 (6)	C8—H8B	0.9600
O5X—C19	1.355 (4)	C8—H8C	0.9600
N1—C18	1.3648 (17)	C9—H9A	0.9600
N1—C10	1.3242 (17)	C9—H9B	0.9600
C1—C2	1.377 (2)	C9—H9C	0.9600
C1—C6	1.4017 (19)	C11—H11A	0.9300
C2—C3	1.405 (2)	C14—H14A	0.9300
C3—C4	1.385 (2)	C15—H15A	0.9300
C4—C5	1.396 (2)	C16—H16A	0.9300
C5—C6	1.395 (2)	C17—H17A	0.9300
C6—C10	1.4852 (19)	C20—H20A	0.9700
C10—C11	1.420 (2)	C20—H20B	0.9700
C11—C12	1.366 (2)	C20X—H20C	0.9700
C12—C19	1.507 (2)	C20X—H20D	0.9700
C12—C13	1.4307 (19)	C21—H21C	0.9600
C13—C18	1.4278 (19)	C21—H21A	0.9600
C13—C14	1.416 (2)	C21—H21B	0.9600
C14—C15	1.368 (2)	C21X—H21D	0.9600
C15—C16	1.405 (2)	C21X—H21E	0.9600
C16—C17	1.366 (2)	C21X—H21F	0.9600
			
C2—O1—C7	115.97 (11)	O1—C7—H7B	109.00
C3—O2—C8	117.06 (11)	O1—C7—H7C	109.00
C5—O3—C9	117.82 (12)	H7A—C7—H7B	109.00
C19—O5—C20	116.5 (3)	H7A—C7—H7C	109.00
C19—O5X—C20X	115.2 (3)	H7B—C7—H7C	110.00
C10—N1—C18	118.72 (12)	O2—C8—H8A	109.00
C2—C1—C6	122.04 (13)	O2—C8—H8B	109.00
O1—C2—C1	125.03 (13)	O2—C8—H8C	109.00
O1—C2—C3	115.89 (12)	H8A—C8—H8B	109.00
C1—C2—C3	119.05 (13)	H8A—C8—H8C	110.00
O2—C3—C2	115.51 (12)	H8B—C8—H8C	109.00
O2—C3—C4	124.83 (13)	O3—C9—H9A	109.00
C2—C3—C4	119.66 (13)	O3—C9—H9B	109.00
C3—C4—C5	120.65 (13)	O3—C9—H9C	109.00
O3—C5—C6	117.12 (12)	H9A—C9—H9B	109.00
C4—C5—C6	120.22 (13)	H9A—C9—H9C	109.00
O3—C5—C4	122.64 (12)	H9B—C9—H9C	110.00
C1—C6—C5	118.19 (13)	C10—C11—H11A	120.00
C5—C6—C10	124.27 (12)	C12—C11—H11A	120.00
C1—C6—C10	117.48 (12)	C13—C14—H14A	120.00
N1—C10—C6	115.55 (12)	C15—C14—H14A	120.00
C6—C10—C11	122.60 (12)	C14—C15—H15A	120.00
N1—C10—C11	121.81 (12)	C16—C15—H15A	120.00
C10—C11—C12	120.52 (13)	C15—C16—H16A	120.00
C11—C12—C13	119.41 (13)	C17—C16—H16A	120.00
C13—C12—C19	121.15 (12)	C16—C17—H17A	120.00
C11—C12—C19	119.44 (12)	C18—C17—H17A	120.00
C12—C13—C14	125.81 (13)	O5—C20—H20A	110.00
C14—C13—C18	118.19 (12)	O5—C20—H20B	110.00
C12—C13—C18	115.97 (12)	C21—C20—H20A	110.00
C13—C14—C15	120.82 (14)	C21—C20—H20B	110.00
C14—C15—C16	120.71 (14)	H20A—C20—H20B	109.00
C15—C16—C17	120.52 (14)	O5X—C20X—H20D	109.00
C16—C17—C18	120.16 (14)	C21X—C20X—H20C	109.00
N1—C18—C13	123.51 (12)	C21X—C20X—H20D	109.00
N1—C18—C17	116.89 (12)	H20C—C20X—H20D	108.00
C13—C18—C17	119.59 (12)	O5X—C20X—H20C	109.00
O4—C19—C12	126.01 (13)	H21A—C21—H21C	109.00
O4—C19—O5X	123.5 (2)	H21B—C21—H21C	110.00
O4—C19—O5	120.22 (19)	C20—C21—H21A	109.00
O5X—C19—C12	108.59 (18)	C20—C21—H21B	109.00
O5—C19—C12	111.91 (18)	C20—C21—H21C	109.00
O5—C20—C21	107.5 (3)	H21A—C21—H21B	110.00
O5X—C20X—C21X	111.1 (4)	C20X—C21X—H21D	109.00
C2—C1—H1A	119.00	C20X—C21X—H21E	109.00
C6—C1—H1A	119.00	C20X—C21X—H21F	110.00
C3—C4—H4A	120.00	H21D—C21X—H21E	109.00
C5—C4—H4A	120.00	H21D—C21X—H21F	109.00
O1—C7—H7A	109.00	H21E—C21X—H21F	109.00
			
C7—O1—C2—C1	14.91 (19)	C4—C5—C6—C10	173.60 (13)
C7—O1—C2—C3	−166.87 (12)	C1—C6—C10—N1	39.18 (18)
C8—O2—C3—C2	−175.94 (12)	C1—C6—C10—C11	−138.53 (14)
C8—O2—C3—C4	4.6 (2)	C5—C6—C10—N1	−137.83 (14)
C9—O3—C5—C4	0.0 (2)	C5—C6—C10—C11	44.5 (2)
C9—O3—C5—C6	178.22 (13)	N1—C10—C11—C12	−0.1 (2)
C20—O5—C19—O4	12.1 (4)	C6—C10—C11—C12	177.43 (13)
C20—O5—C19—C12	177.4 (3)	C10—C11—C12—C13	2.3 (2)
C19—O5—C20—C21	172.6 (3)	C10—C11—C12—C19	−176.45 (13)
C18—N1—C10—C6	−179.53 (12)	C11—C12—C13—C14	175.50 (14)
C18—N1—C10—C11	−1.8 (2)	C11—C12—C13—C18	−2.47 (19)
C10—N1—C18—C13	1.6 (2)	C19—C12—C13—C14	−5.7 (2)
C10—N1—C18—C17	−177.38 (13)	C19—C12—C13—C18	176.29 (12)
C6—C1—C2—O1	−178.50 (13)	C11—C12—C19—O4	162.65 (15)
C6—C1—C2—C3	3.3 (2)	C11—C12—C19—O5	−1.7 (2)
C2—C1—C6—C5	0.4 (2)	C13—C12—C19—O4	−16.1 (2)
C2—C1—C6—C10	−176.77 (13)	C13—C12—C19—O5	179.57 (19)
O1—C2—C3—O2	−1.94 (18)	C12—C13—C14—C15	−178.64 (14)
O1—C2—C3—C4	177.53 (12)	C18—C13—C14—C15	−0.7 (2)
C1—C2—C3—O2	176.39 (12)	C12—C13—C18—N1	0.6 (2)
C1—C2—C3—C4	−4.1 (2)	C12—C13—C18—C17	179.48 (13)
O2—C3—C4—C5	−179.35 (13)	C14—C13—C18—N1	−177.57 (13)
C2—C3—C4—C5	1.2 (2)	C14—C13—C18—C17	1.4 (2)
C3—C4—C5—O3	−179.28 (13)	C13—C14—C15—C16	−0.2 (2)
C3—C4—C5—C6	2.6 (2)	C14—C15—C16—C17	0.5 (2)
O3—C5—C6—C1	178.38 (12)	C15—C16—C17—C18	0.2 (2)
O3—C5—C6—C10	−4.6 (2)	C16—C17—C18—N1	177.90 (13)
C4—C5—C6—C1	−3.4 (2)	C16—C17—C18—C13	−1.1 (2)

### Hydrogen-bond geometry (Å, º)

**Table:**

*D*—H···*A*	*D*—H	H···*A*	*D*···*A*	*D*—H···*A*
C14—H14*A*···O4	0.93	2.30	2.9073 (18)	123
C9—H9*A*···O3^i^	0.96	2.53	3.397 (2)	150
C20—H20*A*···O1^ii^	0.97	2.51	3.304 (5)	139

Symmetry codes: (i) −*x*+1, −*y*+1, −*z*; (ii) *x*−1, *y*+1, *z*.
