# Supplementary figures and images for: Crystal structure of ethyl 2-(2,4,5-tri­meth­oxy­phen­yl)quinoline-4-carboxyl­ate
Source: Acta Crystallogr E Crystallogr Commun. 2015 Jun 27;71(Pt 7):o514–5. doi: 10.1107/S2056989015011706 (PMC4518944; doi:10.1107/S2056989015011706)

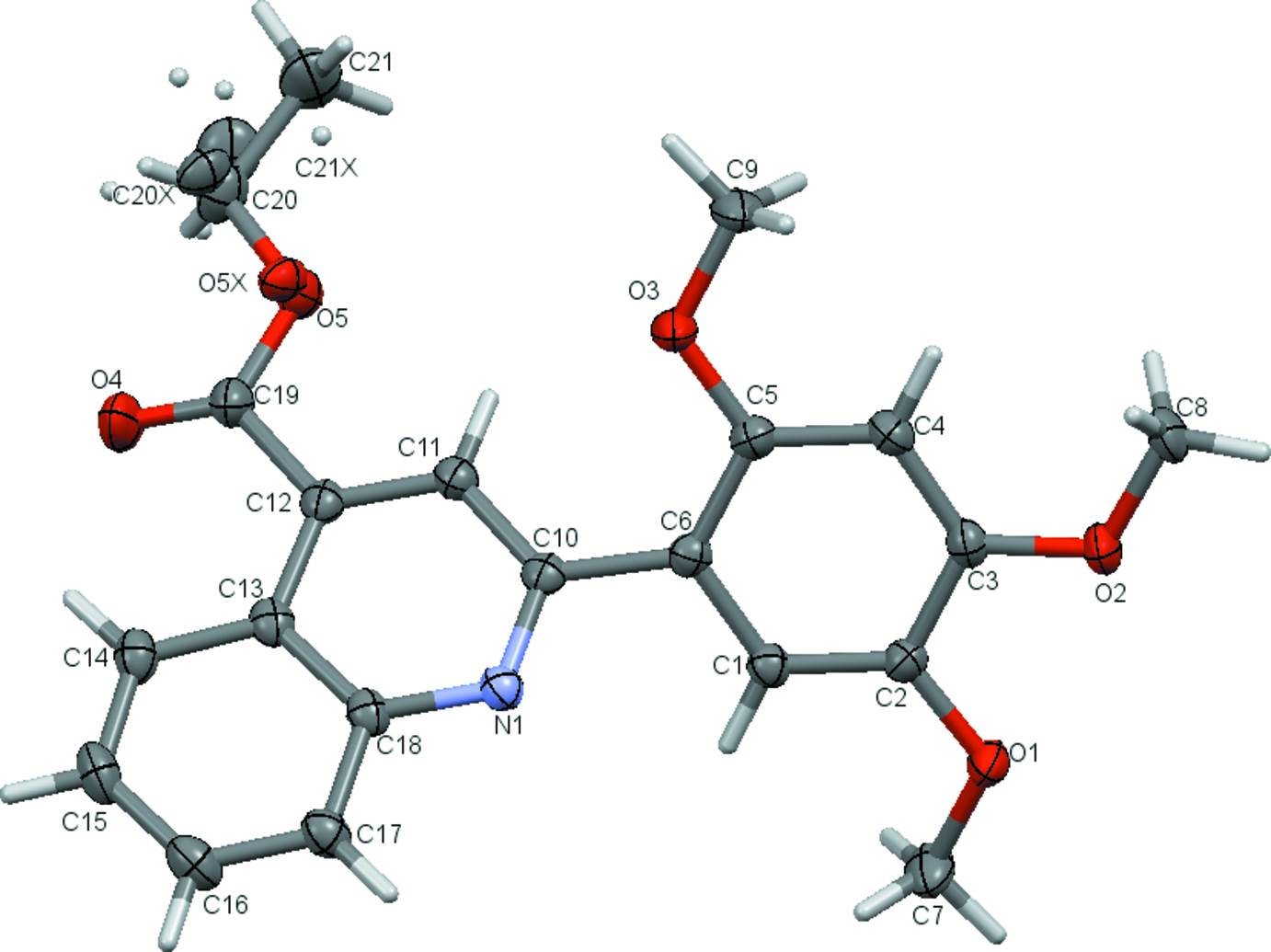

Supplement: Supplementary file 4 [file e-71-0o514-fig1.tif]
